# Differentiating pancreatic neuroendocrine tumors from pancreatic ductal adenocarcinomas by the “Duct-Road Sign”: A preliminary magnetic resonance imaging study

**DOI:** 10.1097/MD.0000000000016960

**Published:** 2019-08-30

**Authors:** Bo Xiao, Zhi-Qiong Jiang, Jin-Xiang Hu, Xiao-Ming Zhang, Hai-Bo Xu

**Affiliations:** a Department of Radiology, Zhongnan Hospital of Wuhan University, Wuhan University, Wuhan; b Sichuan Key Laboratory of Medical Imaging, Department of Radiology; c Department of Geratology, Affiliated Hospital of North Sichuan Medical College, Nanchong, PR China.

**Keywords:** differential diagnosis, duct road sign, MRI, pancreatic ductal adenocarcinomas, pancreatic neuroendocrine tumor

## Abstract

To assess the duct-road sign and tumor-to-duct ratio (TDR) in MRI for differentiating pancreatic neuroendocrine tumors (PNETs) from pancreatic ductal-adenocarcinomas (PDACs).

Retrospectively reviewed MRI characteristics of 78 pancreatic masses (histopathology-proven 25 PNETs and 53 PDACs). Receiver operating characteristics with TDR and diagnostic performance of the duct-road sign for differential diagnosis were performed.

The prevalence of duct-road sign in PNETs was higher than that for PDACs (84% vs 0%; *P* < .001). A strong correlation (r = 0.884, *P* < .001) was observed between MRI for PNETs and the frequency of this sign. Performance characteristics of the duct-road sign in MRI for PNET diagnosis were sensitivity (84%, [21 of 25]), specificity (100%, [53 of 53]), positive predictive value (100%, [21 of 21]), negative predictive value (92.9%, [53 of 57]), and accuracy (94.8%, [74 of 78]). In the intention-to-diagnose analysis, the corresponding values were 67.7% (21 of 31), 100% (53 of 53), 100% (21 of 21), 84.1% (53 of 63), and 88.1% (74 of 84). The TDR in PNETs was observed to be greater than that in PDACs (14.6 ± 9.3 vs 6.9 ± 3.8, *P* = .001). TDR with a cut-off value of 7.7 had high sensitivity (84%) and specificity (66%) with area under curve (0.802, 95% CI: 0.699, 0.904; *P* < .001) for distinguishing PNETs from PDACs.

The presence of duct-road sign and TDR > 7.7 on MRI may assist in diagnosis for PNET instead of PDAC.

## 1 Introduction

Among various pancreatic cancers, pancreatic neuroendocrine tumors (PNETs) account for 2% to 10% of all pancreatic tumor types and are managed via surgery till date.^[1,2]^ Computed tomography (CT) and magnetic resonance imaging (MRI) describe PNETs as highly vascularized and constrained solid masses.^[2]^ On the other hand, pancreatic ductal adenocarcinomas (PDACs) constitute 95% of the total exocrine pancreatic cancers,^[3]^ which are characterized by hypovascularity in CT or MRI. Differential diagnosis of PNETs and PDACs is usually easy due to the differences in tumor margin, vascularization pattern, pancreatic duct dilatation, and pancreatic atrophy among the tumors.^[4]^ However, atypical PNETs with minimal vascularization like the PDACs might present a challenge for the radiologists and lead to misdiagnosis of PNETs and PDACs.^[4–6]^ In addition, the treatment strategies and prognosis of PNETs and PDACs differ in many ways and hence, pretreatment differentiation is worth it to determine the therapeutic strategies.^[4]^

Although CT is the most common modality for identifying enhancement pattern of pancreatic masses, it is unfavorable to appreciate the pancreatic duct system compared with MRI/magnetic resonance cholangiopancreatography (MRCP). MRI has excellent resolution for soft tissues and provides different facilities to enable noninvasive assessment of pancreatic duct and parenchyma, neighboring soft tissues, and vascular network in a single investigation.^[2]^ Previous studies have proposed different signs like “double duct sign”^[7]^ or “interrupted duct sign” ^[8]^ for differential diagnosis of PDACs, after considering morphological features of the main pancreatic duct (MPD). However, unlike PDACs, no specific imaging signs on MPD exist for PNETs (typical and atypical PNETs). Our hypothesis is that the presence of potential signs of the MPD in this setting, particularly in atypical (nonhypervascular) PNETs, which would aid in their differential diagnosis along with preventing misdiagnosis. Thus, the current study was conducted to find potential imaging signs of the MPD in MRI for PNETs.

## 2 Materials and methods

### 2.1 Study design and patient selection

**Figure 1:**
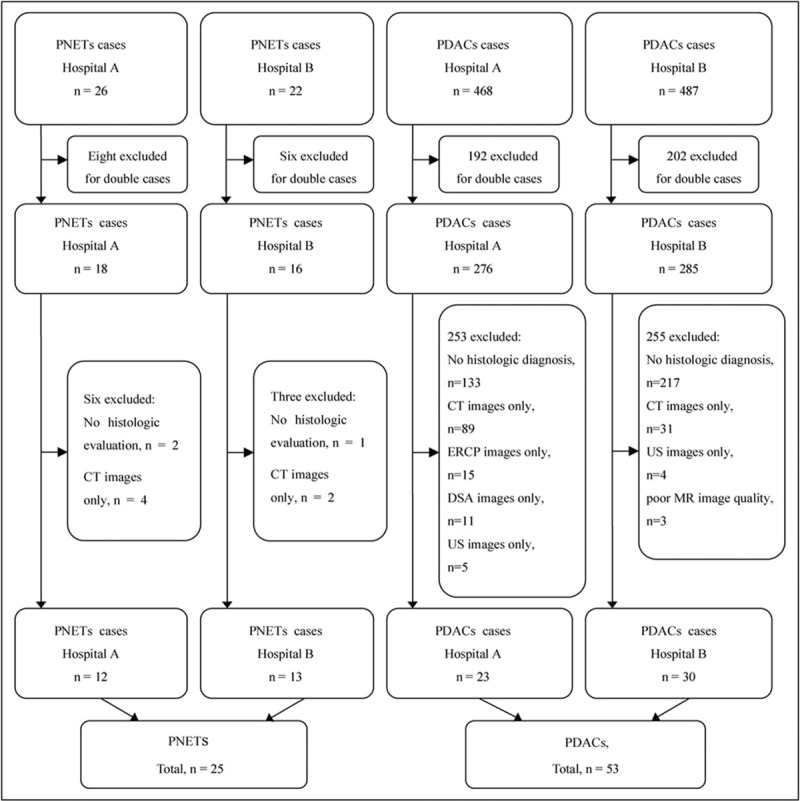
Patient selection flowchart. DSA = digital subtraction angiography, ERCP = endoscopic retrograde cholangiopancreatography, US = ultrasound.

This retrospective study was approved by institutional review boards of our 2 tertiary hospitals. The study was performed in accordance with the Helsinki Declaration of 1983; however, the requirement for informed consent was waived due to the retrospective nature of the study. The radiology databases of the both hospitals were searched for the patients with pancreatic cancer who underwent radiology between January 2010 and January 2018. To include the maximum number of patients, we used search terms such as “pancreatic neuroendocrine tumor,” “islet cell tumor,”’ “insulinoma,” “gastrinoma,” “pancreatic adenocarcinoma,” and “pancreatic cancer.” Using the databases, the study planned to enroll inpatients who underwent abdominal MRI with unenhanced and enhanced phases performed within a month before pancreatectomy. Patients with no histopathological diagnosis of PNETs or PDACs, incomplete clinical records, other imaging examinations such as CT alone, endoscopic retrograde cholangiopancreatography alone, digital subtraction angiography or ultrasound alone, MRI with poor image quality were excluded from the study. Figure 1 presents the selection criteria for patients from both hospitals.

### 2.2 MRI protocols

1.5 T MR scanners were utilized using phased array torso-pelvis coils (GE Medical Systems, Milwaukee, WI) for MRI. To confirm the optimal imaging range of pancreas, coronal T1-weighted scout images were obtained with gradient-echo sequences. Table 1 presents the MR sequences with scanning parameters. Our 2 hospitals shared same abdomen scanning parameters due to same GE scanners. Dynamic contrast-enhanced (DCE) imaging was performed with an axial fat-saturated spoiled gradient-echo (SPGR) sequence. Gadolinium chelate (Schering Guangzhou Co, China) was intravenously administered (0.2 mmol/L per kg body weight) at a dose of 2–3 mL/s by projector injection (Spectris MR Injection System, Medrad Inc, Indianola, PA), followed by a 20-mL saline solution flush. First-pass arterial contrast-enhancement was optimized with a timing bolus sequence (axial SPGR). Dynamic imaging was performed during patient breath-holding before the injection (unenhanced phase), 25 to 30 seconds after the injection (arterial phase), and 55 to 60 seconds after the injection (venous phase).^[9]^

**Table 1:**
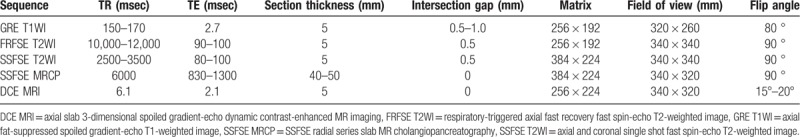
Magnetic resonance imaging parameters.

### 2.3 Duct-road sign—origin and definition

In our previous experiences, we had observed a series of signs in MRI among PNET cases. The observed signs included “a straight road,” “a typical curved road,” and “an atypical curved road,” referring to the MPD passing straight along the edge of a PNET without ductal location displacement (type I), the displaced MPD typically passing around a PNET in the pancreatic body or neck (type II), and only distal or proximal MPD displacement accompanied by tumor location in the head or tail of pancreas (type III), respectively. These morphologic findings all together were named as “duct-road sign” by the authors.

### 2.4 Data analysis

Data analysis was performed using GE healthcare workstations (GE, version: AW 4.6, Sun Microsystems, USA) for MRI data reading and analysis. Two MRI fellowship-trained radiologists (A and B) with > 8 and > 15 years of experience in abdominal MRI performed the data analysis. All other data except the MR images were blinded before evaluation. Both the radiologists reviewed axial and MRCP images independently. In case of discrepancy between the findings of radiologists, a third radiologist (C) with > 25 years clinical MRI experience was consulted and a consensus was reached. Measurements were performed by A based on the consensus findings.

The data recorded for analysis included: site of tumor as head/uncinate, neck, body, or tail, and number of pancreatic tumor. Size of each tumor or each MPD was determined by measuring the largest diameter on axial contrast-enhanced or T2WI images. Final diameter of tumor (D_tumor_) or the MPD (D_duct_) was obtained by taking the mean value of 3 measurements on maximum diameter of each tumor or MPD. Then, a tumor-to-duct ratio (TDR) was quantitatively calculated as D_tumor_/D_duct_. The MPD was considered to be dilated when its diameter was greater than 3 mm. Signal intensity enhancement at postgadolinium images was assessed by subjective reading. Compared with normal pancreatic parenchyma, enhancement features of each tumor at arterial and venous phases were recorded. Typical or traditional PNETs are hypervascular, shown as intense enhancement on imaging. Intense enhance findings of tumor were defined as vivid enhance greater than normal pancreas and approaching enhance of the aorta in the arterial phase, and hyper- or iso-intensity compared with normal pancreas in the venous phase at MRI.^[2,5]^ In contrast, atypical PNETs are nonhypervascular. Therefore, the definite enhancement of tumor may be appreciated in the venous phase rather than the arterial phase.^[2,5]^ When a lesion was heterogeneous on DCE MRI, signal intensity enhancement of predominantly solid component was evaluated. Recording of duct-road sign findings, depicted as aforementioned contents. Lastly, the MRI diagnosis for PNETs or PDACs in the initial MRI reports before surgery was noted. With respect to misdiagnosed patients with PNET (atypical PNETs), the presence or absence of the duct-road sign was reassessed on MRI.

### 2.5 Statistical analysis

All statistical tests were performed using Statistical Package for Social Sciences for Windows (Version 16, Chicago, IL). An analysis of sample size was performed as follows: n’ = [2(μ_α_+μ_β_)^2^P(1−P)]/δ^2^ = [2(1.64+1.28)^2^0.64(1–0.64)]/0.51^2^ = 15.11 ≈ 16. Here, α = 0.05, β = 0.1 (A power of test (1−β) was set as 0.9). Values of *P* and δ (allowable error) were set based on a recent literature 2: when using criteria of a well-circumscribed margin and portal hyper-/isoenhancement, 64% of sensitivity was observed for differential diagnosis of PNETs from PDACs. Thus, the value of *P* was set as .64. Also, in their study, 51% (38 of 74) of PNETs were hypervascular and none of (0 of 82) PDACs were hypervascular. On the basis of their data, the value of δ was set as 0.51 (51%–0%). Moreover, the rate of lost to follow-up was usually set as 20%. Therefore, n = n’ (1+20%) = 16(1+20%) = 19.2 ≈ 20. In another word, the sample size of PNETs in our study should be no less than 20 cases.

The interobserver agreement between 2 reviewers (A and B) for image interpretation in each finding was assessed by using κ statistic for establishing the reliability of interpretation. κ statistic > 0.75, 0.4 to 0.75, and < 0.4 was defined as excellent agreement, fair to good agreement, and poor agreement, respectively. Continuous variables were reported as ranges and mean ± standard deviation. *χ*^2^test or Fisher exact test was applied to determine the significance for difference in categorical variables. Continuous variables in PNETs and PDACs were compared via independent sample *t* test if equal variances assumed (Levene test) or Mann–Whitney *U* test if equal variances not assumed. A receiver operating characteristic (ROC) analysis with the TDR for distinguishing PNETs from PDACs was performed, with diagnostic accuracy evaluated by calculating the area under ROC curve (Az). Az > 0.80 was considered to have good diagnostic accuracy.

A Spearman correlation coefficient (r) was calculated to test the relationship between MRI diagnosis for PNETs and the frequency of appearance of duct-road sign at MRI. The sensitivity, specificity, positive predictive value (PPV), negative predictive value (NPV), and accuracy of the duct-road sign in the broad sense (combined types I, II, and III), in the narrow sense (typical curved road, type II) for distinguishing PNETs from PDACs were calculated by using the standard formulas. Differences in sensitivity, specificity, and accuracy of the duct-road sign, in the narrow sense and in the broad sense, were compared by means of McNemar test. Finally, an analysis of an intention-to-diagnose was also performed. A 2-sided significance level of 0.05 was used for all analyses.

## 3 Results

### 3.1 Baseline characteristics

A total of 48 and 955 consecutive patients with PNET and PDAC were considered initially for evaluation. Of them, 25 (mean age 49.5 ± 11.9 years; 19 females and 6 males) and 53 (mean age 58.6 ± 10.9 years; 34 males and 19 females) consecutive patients with histo-pathologically confirmed PNET and PDAC who underwent gadolinium-enhanced MRI were included in the study. Patients with PNET were significantly younger compared with PDAC patients (*t* = −3.326, *P* = .001). Additionally, PNET group comprised of significantly higher percentage of females compared with PDAC group (76% vs 35.8%; *χ*^2^ = 10.961, *P* = .001).

At baseline, patients with PNET were reported to have undergone surgical procedures, such as pancreaticoduodenectomy (n = 8), distal pancreatectomy without or with splenectomy (n = 12), median pancreatectomy with Roux-en-Y anastomosis (n = 2), and enucleation or segmental pancreatectomy (n = 3). Each of the 25 PNET patients had a solitary lesion. These tumors consisted of 9 with insulinoma, 3 with gastrinoma, and 13 with uncertain types. The PNETs were classified as WHO G1 (n = 14 [56%]), G2 (n = 10 [40%]), and G3 (n = 1 [4%]). On the other hand, PDAC patients underwent pancreatico-duodenectomy (n = 37), distal pancreatectomy and splenectomy (n = 14), and biliary-enterostomy and biopsy of pancreatic head (n = 2). Based on pathology, the PDACs were classified as poorly-differentiated (n = 9), moderate-differentiated (n = 31), and well-differentiated (n = 13).

### 3.2 Tumor location, size, duct dilatation, TDR, and enhancement

The interpretation of MRI for localization of PNETs was excellent by both radiologists (κ ≥ 0.820). The PNETs were located in pancreatic head (n = 6), pancreatic neck (n = 4), pancreatic head and neck (n = 2), pancreatic body (n = 8), pancreatic neck and body (n = 3), and in pancreatic tail (n = 2). PDACs were located in pancreatic head (n = 37), pancreatic neck (n = 2), pancreatic body (n = 10), pancreatic tail (n = 3), and pancreatic body and tail (n = 1).

**Figure 2:**
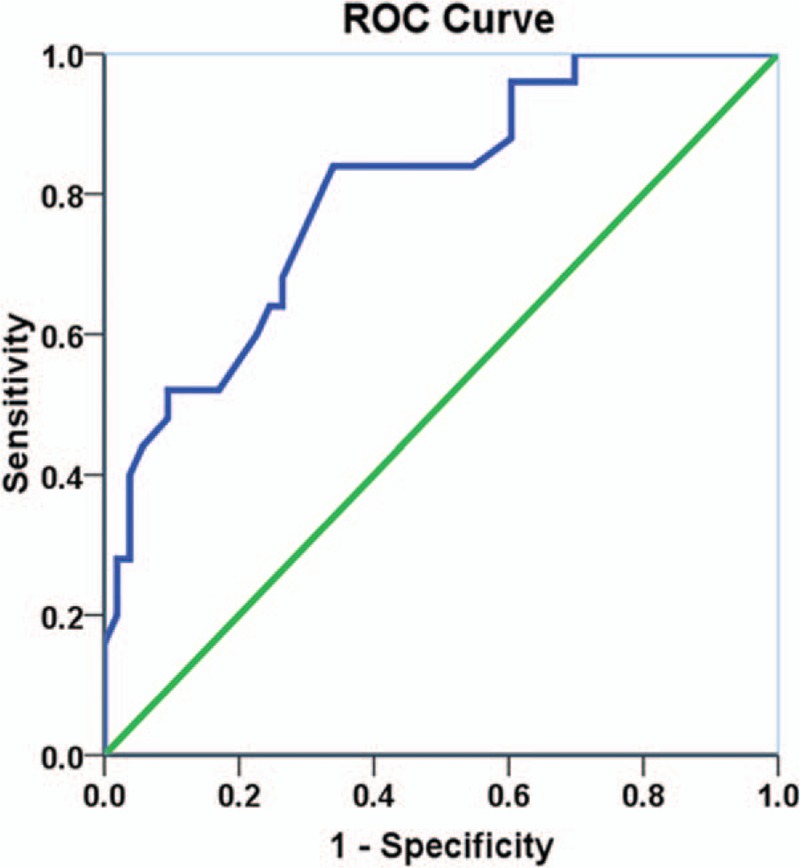
ROC curve with tumor-to-duct ratio (TDR) for differentiating PNETs from PDACs in MRI. MRI = magnetic resonance imaging, PDAC = pancreatic ductal adenocarcinoma, PNET = pancreatic neuroendocrine tumor, ROC = receiver operating characteristic.

The difference in tumor size of PNETs and PDACs was nonsignificant (3.1  cm ± 2.3 cm vs 2.9  cm ± 0.9 cm; *t* = 0.482, *P* = .634). The maximum diameter of MPD in PDACs was significantly higher compared with PNETs (5.6  mm ± 3.5 mm vs 2.4  mm ± 1.2 mm; Z = −5.861, *P* < .001). The incidence of MPD dilatation in MRI of PDAC patients (81.1%, n = 43) was significantly higher than observed for PNET patients (16%, n = 4) (*χ*^2^ = 30.091, *P* < .001). Mean TDR in PNET patients was observed to be greater compared with that in PDAC patients (14.6 ± 9.3 vs 6.9 ± 3.8; Z = 3.906, *P* = .001). For accurate diagnosis of PNET and distinguishing from PDAC, the ROC analysis with TDR on images showed a sensitivity of 84% and a specificity of 66%, with a cut-off value of TDR as 7.7. Area under the ROC curve (Az) was 0.802 (95% CI: 0.699, 0.904; *P* < .001) for distinguishing PNETs from PDACs (Fig. 2). However, there was not statistical difference on TDR in lower grade PNETs (WHO G1) and high grade PNETs (WHO G2 and G3) (14.8 ± 11.1 vs 13.9 ± 6.7; *t* = 0.228, *P* = .821).

For interpretation of tumor enhancement, the κ value for PNETs was 0.737 at MRI between the 2 reviewers. Typical or hypervascular PNETs, shown as intense enhancement in the arterial phase on MRI, were in majority of patients (72%, n = 18). On the other hand, atypical or nonhypervascular PNETs were in the remaining patients (28%, n = 7), with heterogeneous lesions and definite enhancement appreciated in the venous phase rather than arterial phase. For patients with PDAC, none of tumors were hypervascular in the arterial phase, determined as venous phase delayed enhancement on MRI.

### 3.3 MRI for the duct-road sign

**Figure 3:**
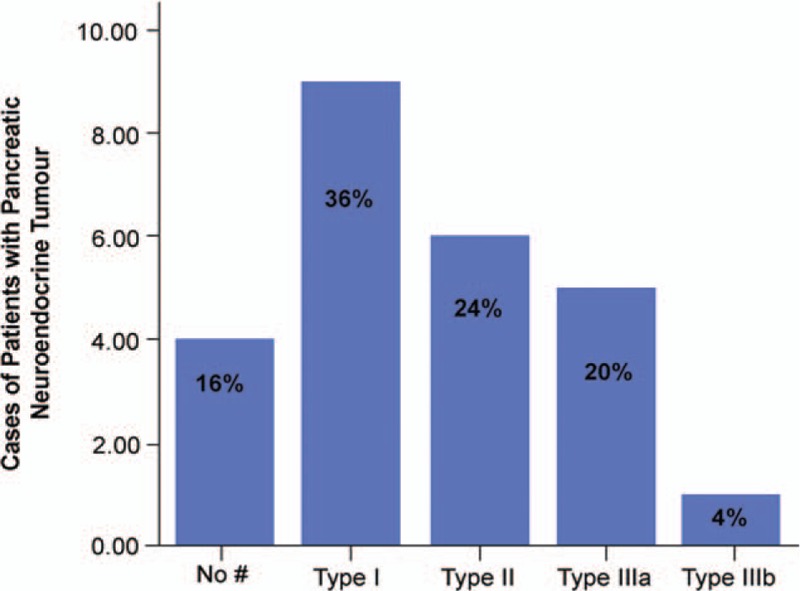
Proportion of PNET patients with different duct-road sign categories. ^#^ without each type of duct-road sign at MR imaging in PNET patients. MR = magnetic resonance, PNET = pancreatic neuroendocrine tumor.

**Figure 4:**
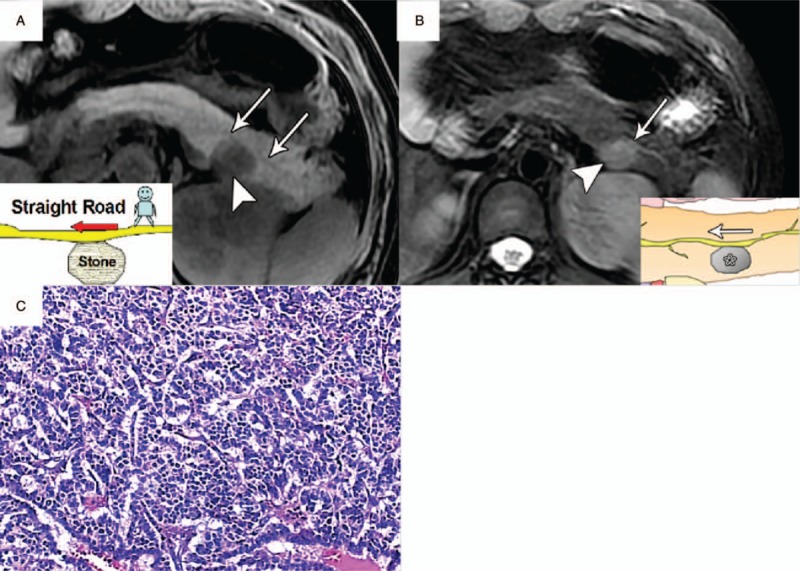
PNET (WHO grade 1) in a 40-year-old woman with type I duct-road sign (straight road sign). A, Axial T1-weighted MR image showed a hypointense tumor (arrowhead) in the pancreatic body with a maximum diameter of 2.4 cm. The pancreatic duct demonstrated normal-caliber size (arrows) with duct tangency by the edge of tumor, and tumor-to-duct ratio was 12. B, Axial T2-weighted MR image showed a hyperintense tumor (arrowhead) with pancreatic duct (arrow) passing straight along the edge of tumor without ductal displacement. C, Photomicrograph sample shows a well-differentiated endocrine neoplasm with stromal fibrosis. (×100, hematoxylin-eosin [H–E] stain). MR = magnetic resonance, PNET = pancreatic neuroendocrine tumor.

**Figure 5:**
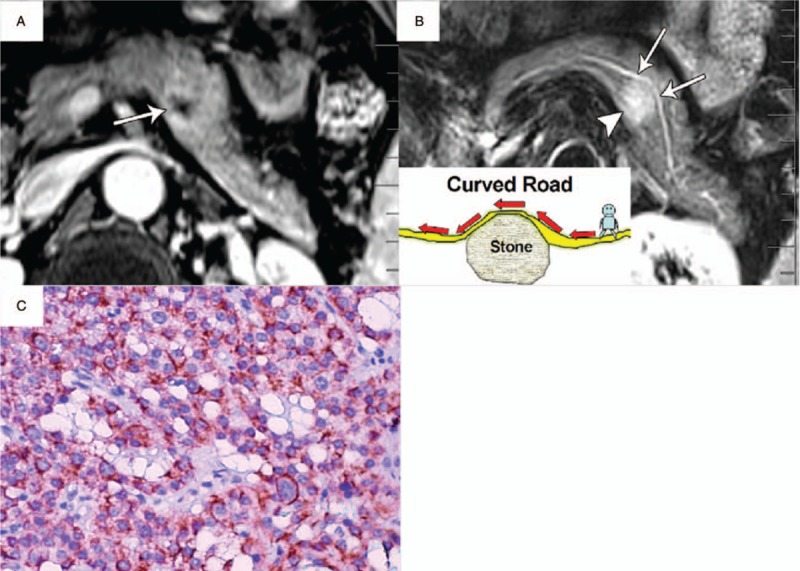
PNET (WHO grade 1) with type II duct-road sign (typical curved road sign) in a 55-year-old man. A, Axial postcontrast MR scan showed an inhomogeneous enhancing tumor (arrow) in the pancreatic body. B, Axial T2-weighted MR image showed a hyperintense tumor (arrowhead) with a maximum diameter of 1.9 cm. The pancreatic duct with a diameter of 2 mm demonstrated passing around the edge of tumor (typical curved road sign) and obvious location displacement (arrows). The tumor-to-duct ratio was 9.5. C, Photomicrograph sample showed positive chromogranin A (CgA) immunoreactivity of neoplastic cells. (×100, immunohistochemical stain). MR = magnetic resonance, PNET = pancreatic neuroendocrine tumor.

**Figure 6:**
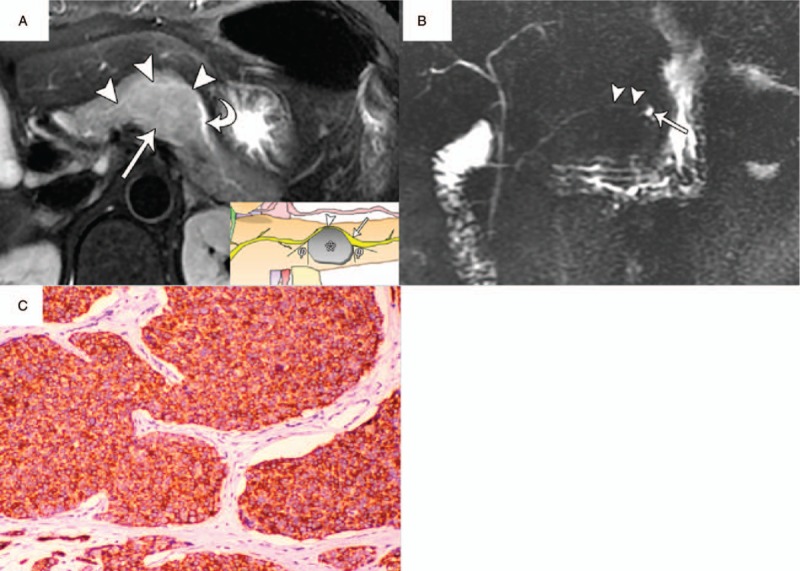
PNET (WHO grade 2) in a 44-year-old woman with type II duct-road sign (typical curved road sign). A, Axial T2-weighted MR image showed a relatively hyperintense tumor (arrow) with a maximum diameter of 3.3 cm in the pancreatic body. The mildly distended pancreatic duct (curved arrow) with a diameter of 3.5 mm demonstrated passing around the edge of tumor and typical curved road sign (arrowheads). The tumor-to-duct ratio was 9.4. B, MRCP showed distal dilatated duct (arrow), as well as the preserved continuity of whole MPD (arrowhead). C, Photomicrograph sample showed positive synaptophysin (Syn) immunoreactivity of neoplastic cells. (×100, immunohistochemical stain). MPD = main pancreatic duct, MRCP = magnetic resonance cholangiopancreatography, MR = magnetic resonance, PNET = pancreatic neuroendocrine tumor.

**Figure 7:**
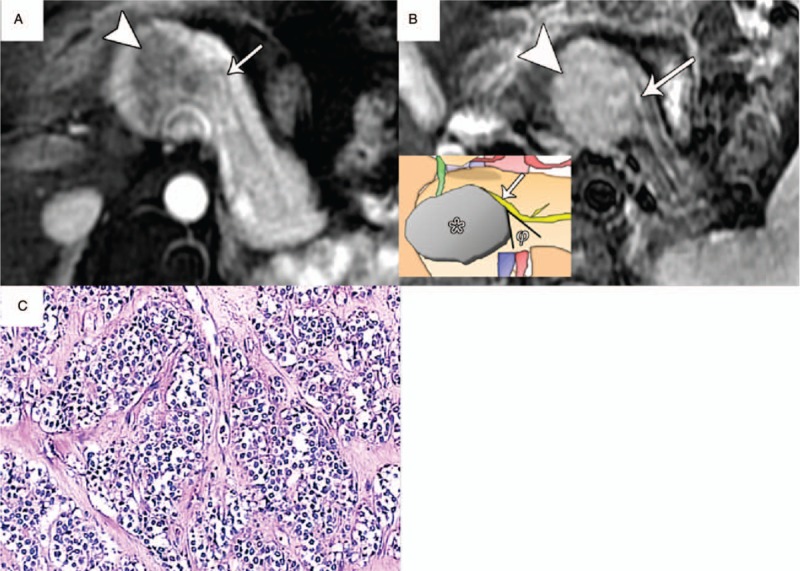
Pancreatic islet cell tumor (WHO grade 1) in a 26-year-old woman with type IIIa duct-road sign (atypical curved road sign). A, Axial postcontrast arterial phase MR image showed a hypovascularity tumor (arrowhead) with a maximum diameter of 3.5 cm in the pancreatic head that can closely mimic a pancreatic adenocarcinoma. Importantly, the pancreatic duct (arrow) was obviously pushed by this tumor and seen as no dilatation finding with a diameter of 2 mm. And then the tumor-to-duct ratio was 17.5. B, Axial T2-weighted MR image showed a hyperintense tumor (arrowhead) with the displaced distal MPD alone (arrow) instead of duct obstruction or cut-off sign. An acute angle was observed between the displaced pancreatic duct and corresponding side of tumor. C, Photomicrograph sample showed positive synaptophysin (Syn) immunoreactivity of neoplastic cells. (×100, immunohistochemical stain). MPD = main pancreatic duct.

**Figure 8:**
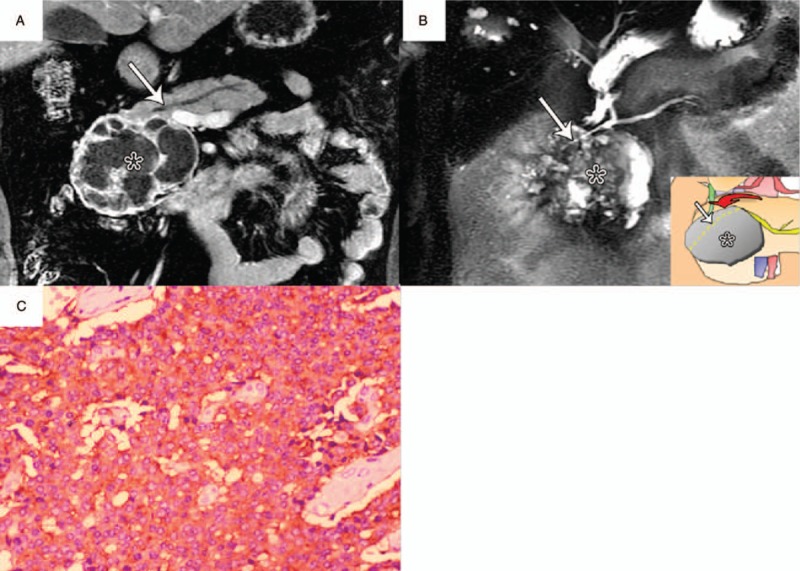
Nonsyndromic PNET (WHO grade 1) in a 44-year-old man with type IIIa duct-road sign (atypical curved road sign). A, Coronal postcontrast venous phase MR image showed a 9.3-cm-diameter heterogeneous tumor (∗) in pancreatic head zone that caused mildly dilated MPD (arrow) with a diameter of 4 mm. And then the tumor-to-duct ratio was 23.8. B, MRCP image showed a relatively hyperintense tumor (∗) with the compressed distal MPD (arrow) passing around finding (atypical curved road sign). An acute angle was observed between displaced pancreatic duct and corresponding side of tumor. C, Photomicrograph sample showed positive synaptophysin (Syn) immunoreactivity of neoplastic cells. (×100, immunohistochemical stain). MPD = main pancreatic duct, MRCP = magnetic resonance cholangiopancreatography, MR = magnetic resonance, PNET = pancreatic neuroendocrine tumor.

Among all 25 patients with PNET, findings of the duct-road sign at MRI were detected in 21 patients (84%). The interpretation of duct-road sign of PNETs was excellent by 2 radiologists (κ ≥ 0.910). The occurrence of duct-road sign at MRI (Fig. 3) showed PNET patients with type I with greatest frequency, by decreasing order as type II, followed by type IIIa, unspecified type (without duct-road sign) and type IIIb. For the duct-road sign of type I (the straight road sign), the MPD on axial T2-weighted MR images demonstrated passing straight along the edge of tumor without ductal location displacement (Fig. 4). For type II duct-road sign (typical curved road sign), the MPD at axial T2-weighted images was apparently pushed by a tumor in the pancreatic body or neck, but the continuity of whole MPD was preserved (Fig. 5). And the diameter of MPD remained normal (Fig. 5) or mild dilatation in appearance (Fig. 6). With respect to type III duct-road sign (atypical curved road sign), the MPD on axial T2-weighted MRI (Fig. 7) or MRCP (Fig. 8) presented as either distal or proximal duct displacement in relation to the tumor located in the pancreatic head (type IIIa) or tail (type IIIb). Moreover, the tumor size and relevant TDR value of PNETs in the different type of duct-road sign on MRI were shown in Table 2.

**Table 2:**
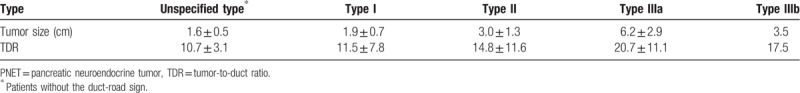
Tumor average size and tumor-to-duct ratio of PNETs (N = 25) in the different form of the duct-road sign on MRI.

**Figure 9:**
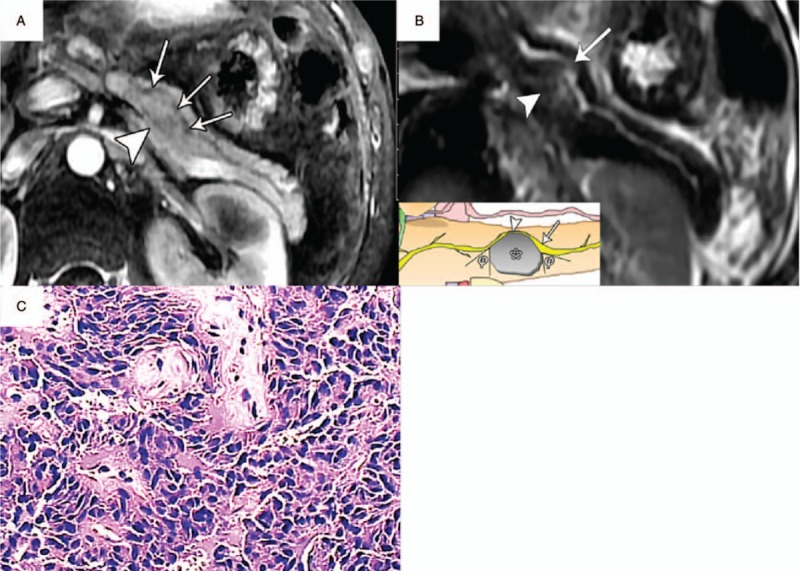
PNET (WHO grade 1) in a 37-year-old man with imaging misdiagnosis as PDAC. A, Axial postcontrast venous phase MR image showed a heterogeneous enhancing tumor (arrowhead) with a maximum diameter of 2.6 cm in the pancreatic body. The pancreatic duct was mildly dilatated (arrows). These findings formed imaging misdiagnosis as a PDAC. B, Axial T2-weighted MR image showed a minimally hyperintense tumor (arrowhead) in the pancreatic body. However, the pancreatic duct was compressed by this tumor and formed the duct-road sign of type II (typical curved road sign) (arrows) rather than duct cut-off finding. The distal pancreatic duct was 4 mm in diameter and tumor-to-duct ratio was 6.5. C, Photomicrograph sample shows a well-differentiated endocrine neoplasm with marked stromal fibrosis. (×100, hematoxylin-eosin [H–E] stain). PDAC = pancreatic ductal adenocarcinoma, PNET = pancreatic neuroendocrine tumor, MR = magnetic resonance.

**Figure 10:**
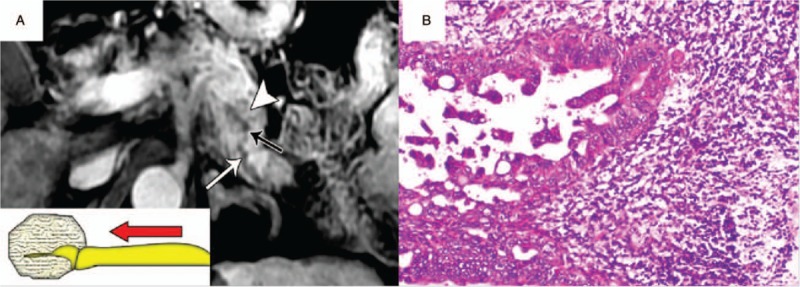
Small PDAC in a 60-year-old man without the duct-road sign. A, Axial postcontrast venous phase MR image showed a small hypoenhanced tumor (arrowhead) with a maximum diameter of 1.8 cm in the pancreatic body. Although this intraparenchymal neoplasm was less than 2 cm in diameter, it resulted in the MPD obvious dilatation (a diameter of 5.5 mm) (white arrow) in pancreatic tail and an interrupted duct sign (black arrow), rather than straight duct-road sign or curved road sign. The tumor-to-duct ratio was only 3.3. B, Photomicrograph of a specimen showed a moderately differentiated ductal adenocarcinoma. (×40, hematoxylin-eosin [H–E] stain). MPD = main pancreatic duct, MR = magnetic resonance, PDAC = pancreatic ductal adenocarcinoma.

In the initial MRI reports, we made the correct diagnosis for 72% (n = 18) of cases with PNET, which was premised on the said traditional imaging evaluation of PNETs (hypervascular or intense enhancement). Among 7 atypical PNET patients, 5 cases who had heterogeneous tumor with lesion enhancement definitively appreciated in venous phase were misdiagnosed as PDAC in initial MRI reports (the remaining 2 patients with uncertain diagnosis on initial reports). Interestingly, the duct-road sign registered in all of the 5 misdiagnosed cases based upon MR images review. Of them, MRI of 3 patients showed the MPD morphologic changes as type II duct-road sign (Fig. 9), instead of cutoff or interrupted duct sign. In other 2 patients, MRI appearances of MPD were the duct-road sign of type IIIa (Fig. 7) without ductal interrupted finding. However, the duct-road sign was not seen in aforementioned 2 patients with uncertain diagnosis on initial MRI reports. On the other hand, none of PDAC patients had MRI features of duct-road sign. Instead, traditional imaging findings on marked obstruction and the interrupted duct sign or cut-off sign at tumor lesion site were demonstrated among PDACs, even though the tumor was smaller with a maximum diameter ≤ 2 cm (Fig. 10).

### 3.4 Frequency and validation of duct-road sign for PNET

The frequency of occurrence of duct-road sign was significantly higher in PNETs compared with PDACs (*χ*^2^ = 60.922, *P* < .001). In addition, a significant correlation (r = 0.884, *P* < .001) was observed between MRI diagnosis for PNET group and the frequency of duct-road sign on MRI. Furthermore, Table 3 presents the validation analysis for PNET compared with PDAC with all types of duct-road sign. McNemar test revealed that the sensitivity (*χ*^2^ = 1.504, *P* < .001) and accuracy (*χ*^2^ = 1.504, *P* < .001) for duct-road sign in the broad sense (combined types I, II, and III) were significantly higher than those in the narrow sense (typical curved road sign), although there was no difference (*P* > .05) in specificity between duct-road sign in the broad sense and in the narrow sense. When considering the subjects in whom the sample was not or the PNET lesion was not seen at MRI (n = 6 [CT performed only]; intention-to-diagnose analysis), the values of performance of duct-road sign in the diagnosis for PNET and distinguishing from PDAC were sensitivity, 67.7% (21 of 31); specificity, 100% (53 of 53); PPV, 100% (21 of 21); NPV, 84.1% (53 of 63); and accuracy, 88.1% (74 of 84).

**Table 3:**
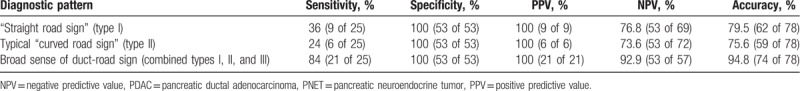
Diagnostic performance in the duct-road sign for PNETs compared with PDACs.

## 4 Discussion

PNETs can imitate PDACs in some instances, since PNETs may present relatively hypoenhanced pattern in the arterial phase and heterogeneous enhancement in the venous phase on postcontrast MRI. It may lead to misdiagnosis.^[10,11]^ Therefore, it would be of great utility if we can accurately discriminate PNETs from PDACs using some other potential imaging markers. In the present study, we observed that the duct-road sign was common among patients with PNET on MRI. The frequency of occurrence of duct-road sign in MRI was significantly higher (*P* < .001) in PNETs compared with PDACs. A positive correlation (r = 0.884, *P* < .001) was observed between MRI diagnosis for PNET and the prevalence of duct-road sign at images. Moreover, the duct-road sign also registered in our misdiagnosed atypical/nonhypervascular PNET patients. Thus, the duct-road sign may be used as a simple and supplementary imaging sign to the traditional imaging findings for the purpose of distinguishing PNETs from PDACs.

The morphological features of pancreatic duct are of great value for the diagnosis and differential diagnosis on pancreatic and related carcinomas.^[7,8,12,13]^ A prior study reported the simultaneous dilatation of biliary and pancreatic duct was seen as a “double duct sign” for the differential diagnosis of periampullary carcinomas.^[13]^ Kim et al^[7]^ found that combined double duct sign and duct obstruction were common in patients with pancreatic carcinomas, which appeared like a 4-segment sign. Similar types of duct obstruction were observed in our study as well among patients of PDAC. Further to add to the evidence, Prokesch et al^[8]^ reported an interrupted duct sign as one of important indicators for the presence of iso-attenuating PDAC. A retrospective study by Ichikawa et al^[12]^ described that a duct penetration sign was in 85% of inflammatory pancreatic mass patients, compared with 4% in pancreatic carcinoma patients. Unlike these signs, there are no specific ductal signs at images for differential diagnosis of PNETs. In the present study, the duct-road sign was observed in 84% of our PNET patients, whereas none of PDAC patients had the sign in MRI. In addition, the duct-road sign, as a criterion for PNET, had a sensitivity of 84% and specificity of 100% for the purpose of differentiating PNETs from PDACs in a broad sense. If the duct-road sign was defined as “typical curved road sign” of type II in the narrow sense, the specificity was also 100%. Furthermore, when the enhancement of PNET is atypical (heterogeneous and nonhypervascular on MRI), the duct-road sign could also be considered a valuable indirect feature in this setting. Consequently, we recommend this new imaging sign—duct-road sign—like “different pathways of the MPD” which may be a helpful sign correlating PNETs instead of PDACs.

Pancreas is mainly composed of the pancreatic parenchyma and the MPD.^[14]^ PDACs typically arise from the MPD, whereas PNETs derive from the pancreatic parenchyma.^[15]^. MPD dilatation is correlated to both PDACs and PNETs but more likely associated with PDACs.^[14–17]^ A prior study reported that a significant (*P* < .01) increase in the MPD dilatation and minimal decrease in mean tumor size emerged among the PDAC patients, compared with PNET patients.^[2]^ Another study described that increased MPD dilatation was in PDAC patients; however, nonsignificant (*P* > .05) difference was identified on the ductal dilatation incidence between PDACs and PNETs. Also, very slight decrease in the mean tumor size was observed in PDACs compared with PNETs.^[4]^ In agreement with the previous studies, we also observed increased MPD dilatation and decreased mean tumor size presented among PDACs compared with PNETs. In the current study, we also obtained another new indicator—significantly increased TDR (mean TDR value more than 7.7) in the PNET patients, which was due to increased tumor size and decreased MPD dilatation in PNETs. Thus, the TDR analysis with a cut-off point of 7.7 may be considered one of the predictive markers in the differential diagnosis for PNETs, as it showed a favorable specificity and sensitivity in ROC analysis.

Recently, Kim et al^[18]^ reported characteristics of PNETs as common and uncommon CT findings according to grade of tumors. The common CT findings included well-circumscribed, homogeneous, hypervascular, and hyperintense tumor enhancement in lower grade PNETs (WHO G1). And the uncommon CT findings included less-defined, heterogeneous, hypovascular, hypointense tumor enhancement in high-grade PNETs (WHO G2 and G3).^[18]^ Consistent with the results, we also observed ill-defined, heterogeneous, hypovascular, and less intense tumor enhancement in a minority of our PNET cases. However, in our study, we also obtained there was not statistical difference on mean TDR value between lower grade PNETs (WHO G1) and high grade PNETs (WHO G2 and G3). This phenomenon is probably responsible for no significant difference on mean tumor size between lower grade and high grade PNETs, as well as the similar diameter of MPD among different grades of tumors in our PNET cases.

Our study had several limitations. First, the present findings could not indicate the absolute sensitivity, specificity, or accuracy of the presence of duct-road sign on MRI for the prospective diagnosis of PNET, because this study did not include any other kinds of pancreatic neoplasms. Second, our study included limited sample size, since PNET is a rare pancreatic tumor with an annual incidence of 0.19 to 0.32/100,000.^[1]^ It has been reported that some very rare PNETs even with small size can secrete serotonin, which results in fibrotic structuring of the MPD, ductal obstruction, and upstream ductal dilatation.^[19,20]^ Therefore, we suggest that there is a limitation for this sign because the MPD change in those rare PNETs may be similar to related features of PDACs. Third, the study coordinator provided the standard of the duct-road sign for reviewing the MR images, which might produce incorporation bias and result in an overestimation of accuracy. Hence, further prospective multicenter studies may be needed to validate clinical implications of the duct-road sign in larger populations and patients with other kinds of pancreatic tumors.

## 5 Conclusion

In conclusion, the duct-road sign is frequently observed in MRI among PNET patients. The duct-road sign closely correlates with the differential diagnosis of PNET and it may be beneficial in differentiating PNETs from PDACs besides the traditional imaging findings. Also, the TDR analysis with a point > 7.7 in MRI may be considered as the diagnosis for PNET rather than PDAC.

## Author contributions

**Conceptualization:** Bo XIAO, Jinxiang Hu, Haibo Xu.

**Data curation:** Bo XIAO, Zhiqiong Jiang, Jinxiang Hu, Xiaoming Zhang.

**Formal analysis:** Jinxiang Hu, Haibo Xu.

**Funding acquisition:** Haibo Xu.

**Investigation:** Bo XIAO, Zhiqiong Jiang, Jinxiang Hu, Xiaoming Zhang, Haibo Xu.

**Methodology:** Bo XIAO.

**Resources:** Bo XIAO, Zhiqiong Jiang, Jinxiang Hu, Xiaoming Zhang.

**Software:** Bo XIAO, Zhiqiong Jiang.

**Supervision:** Haibo Xu.

**Validation:** Bo XIAO, Xiaoming Zhang.

**Visualization:** Bo XIAO, Zhiqiong Jiang, Xiaoming Zhang.

**Writing – original draft:** Bo XIAO.

**Writing – review & editing:** Bo XIAO, Haibo Xu.

Bo XIAO orcid: 0000-0001-5862-974X.
